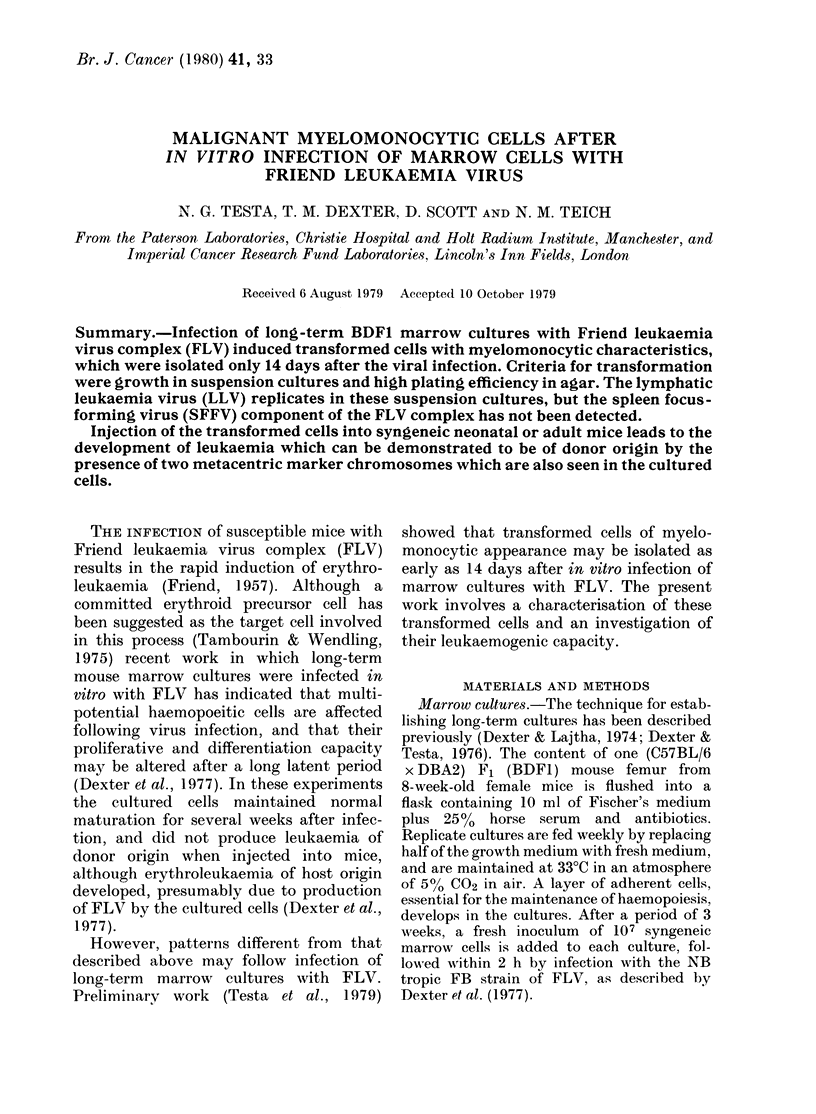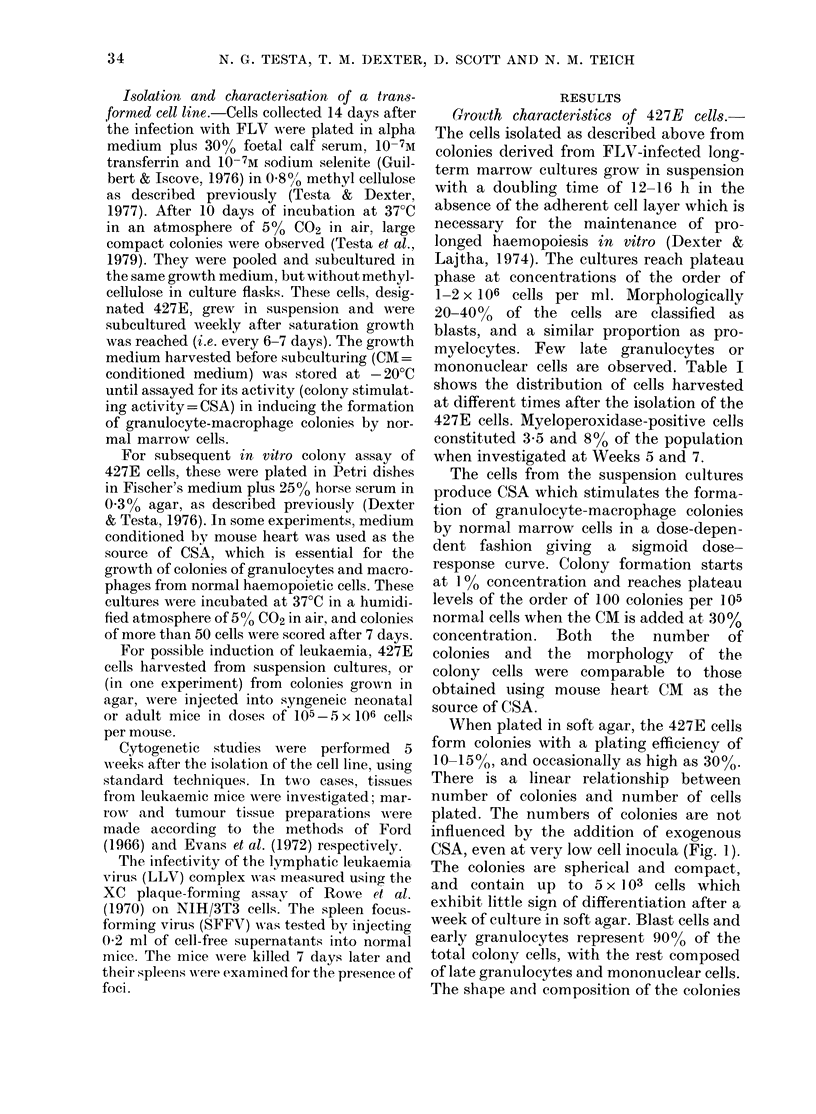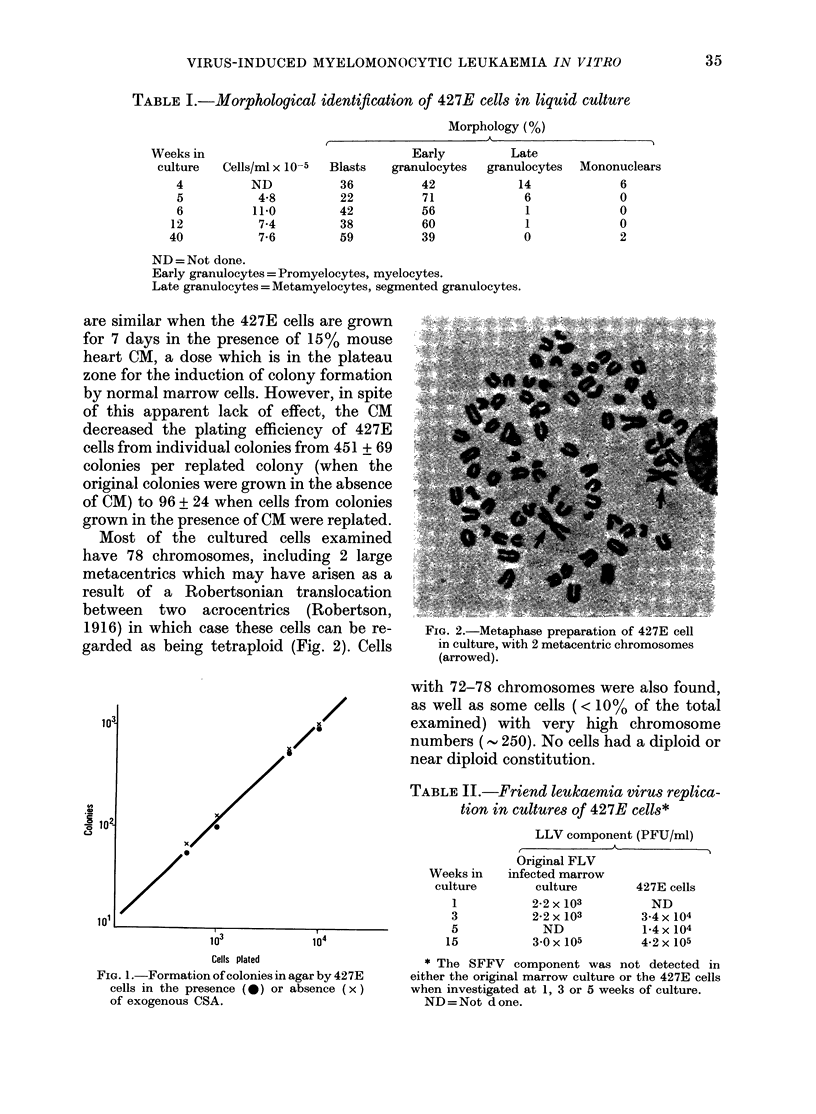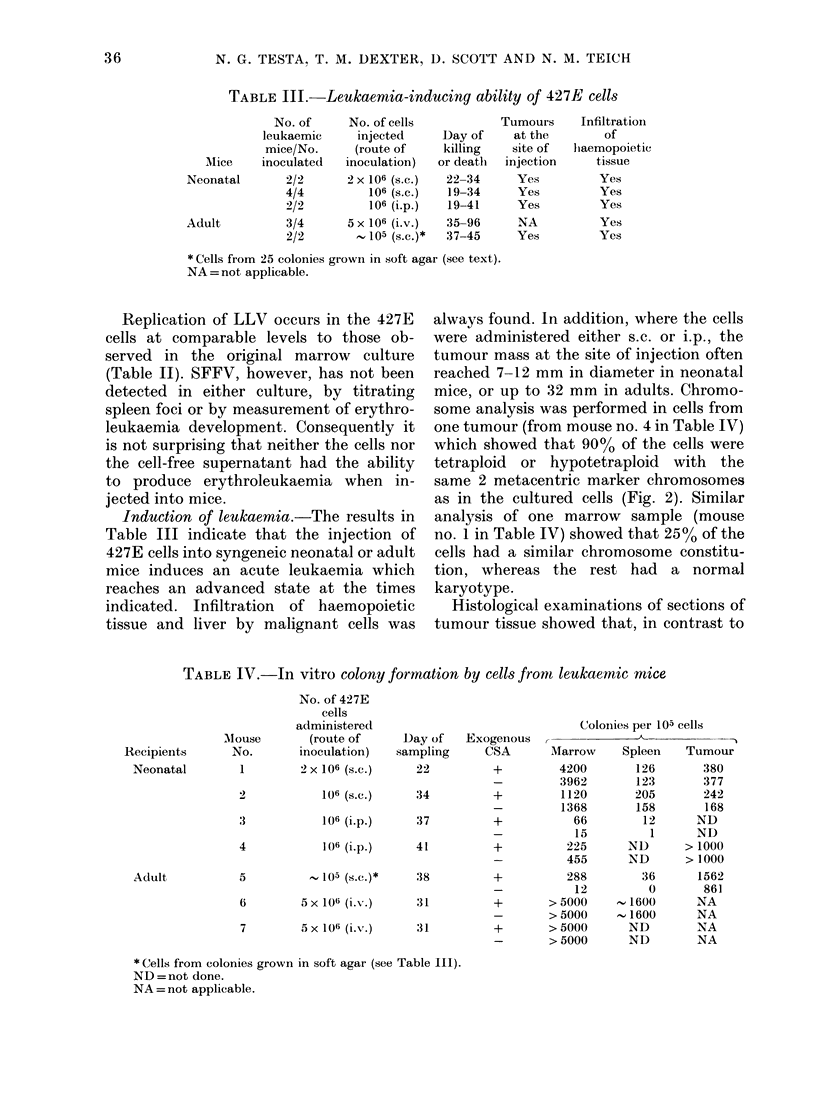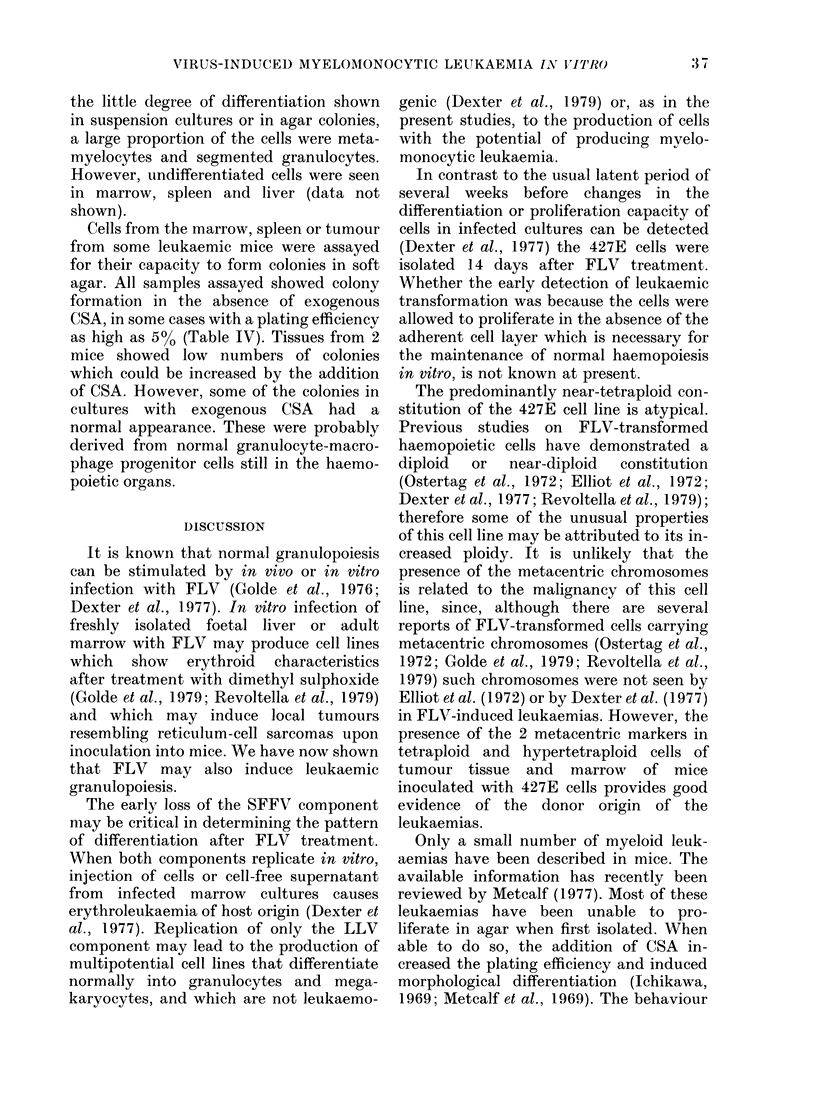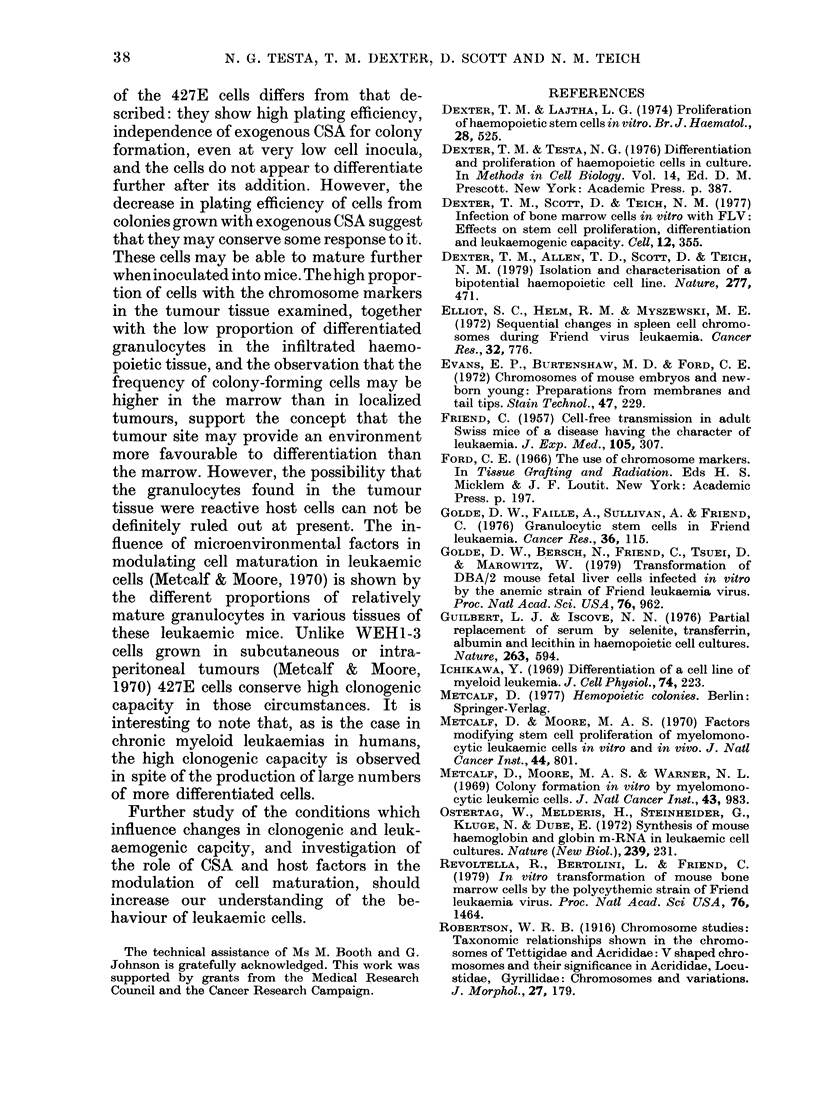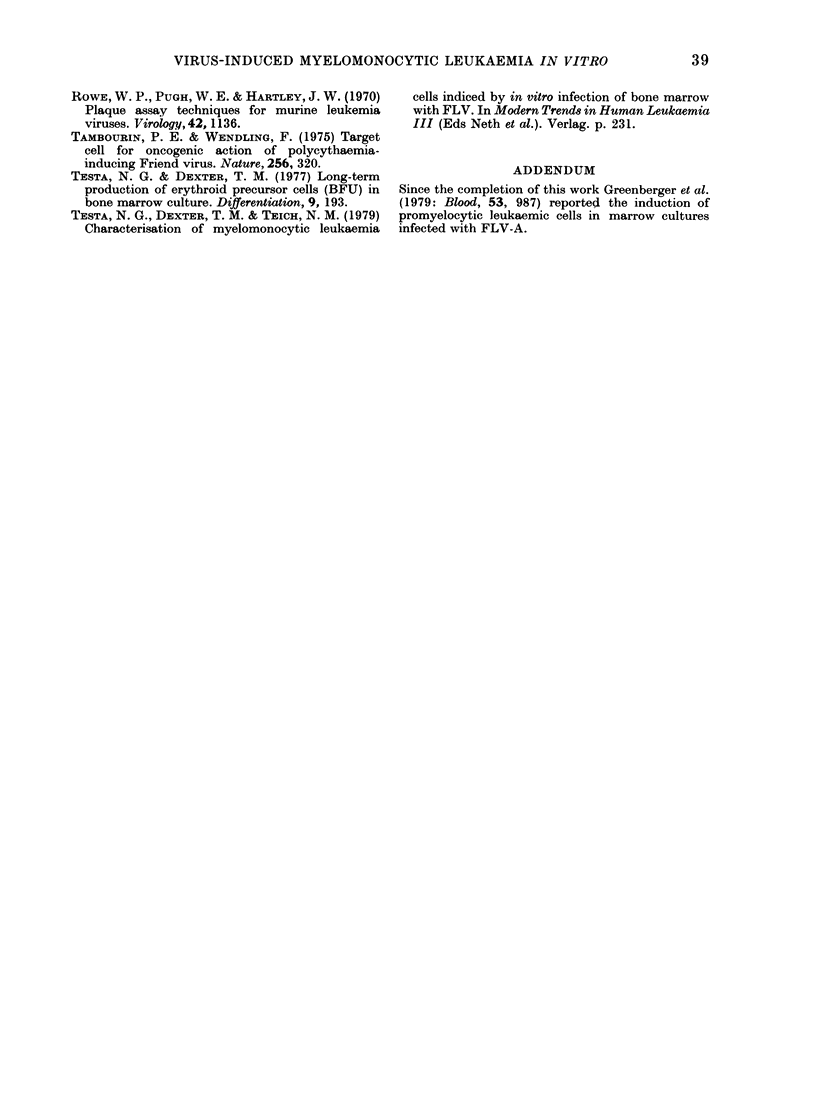# Malignant myelomonocytic cells after in vitro infection of marrow cells with Friend leukaemia virus.

**DOI:** 10.1038/bjc.1980.4

**Published:** 1980-01

**Authors:** N. G. Testa, T. M. Dexter, D. Scott, N. M. Teich

## Abstract

**Images:**


Br. J. Cancer (1980) 41, 33

### MALIGNANT MYELOMONOCYTIC CELLS AFTER *IN VITRO* INFECTION OF MARROW CELLS WITH FRIEND LEUKAEMIA VIRUS

N. G. TESTA, T. M. DEXTER, D. SCOTT AND N. M. TEICH

*From the Paterson Laboratories, Christie Hospital and Holt Radium Institute, Manchester, and Imperial Cancer Research Fund Laboratories, Lincoln's Inn Fields, London*

Received 6 August 1979 Accepted 10 October 1979

**Summary.—Infection of long-term BDF1 marrow cultures with Friend leukaemia virus complex (FLV) induced transformed cells with myelomonocytic characteristics, which were isolated only 14 days after the viral infection. Criteria for transformation were growth in suspension cultures and high plating efficiency in agar. The lymphatic leukaemia virus (LLV) replicates in these suspension cultures, but the spleen focus-forming virus (SFFV) component of the FLV complex has not been detected.**

**Injection of the transformed cells into syngeneic neonatal or adult mice leads to the development of leukaemia which can be demonstrated to be of donor origin by the presence of two metacentric marker chromosomes which are also seen in the cultured cells.**

THE INFECTION of susceptible mice with Friend leukaemia virus complex (FLV) results in the rapid induction of erythroleukaemia (Friend, 1957). Although a committed erythroid precursor cell has been suggested as the target cell involved in this process (Tambourin & Wendling, 1975) recent work in which long-term mouse marrow cultures were infected *in vitro* with FLV has indicated that multipotential haemopoeitic cells are affected following virus infection, and that their proliferative and differentiation capacity may be altered after a long latent period (Dexter *et al.*, 1977). In these experiments the cultured cells maintained normal maturation for several weeks after infection, and did not produce leukaemia of donor origin when injected into mice, although erythroleukaemia of host origin developed, presumably due to production of FLV by the cultured cells (Dexter *et al.*, 1977).

However, patterns different from that described above may follow infection of long-term marrow cultures with FLV. Preliminary work (Testa *et al.*, 1979) showed that transformed cells of myelomonocytic appearance may be isolated as early as 14 days after *in vitro* infection of marrow cultures with FLV. The present work involves a characterisation of these transformed cells and an investigation of their leukaemogenic capacity.

### MATERIALS AND METHODS

*Marrow cultures.*—The technique for establishing long-term cultures has been described previously (Dexter & Lajtha, 1974; Dexter & Testa, 1976). The content of one (C57BL/6 ×DBA2) $F_1$ (BDF1) mouse femur from 8-week-old female mice is flushed into a flask containing 10 ml of Fischer's medium plus 25% horse serum and antibiotics. Replicate cultures are fed weekly by replacing half of the growth medium with fresh medium, and are maintained at 33°C in an atmosphere of 5% $CO_2$ in air. A layer of adherent cells, essential for the maintenance of haemopoiesis, develops in the cultures. After a period of 3 weeks, a fresh inoculum of $10^7$ syngeneic marrow cells is added to each culture, followed within 2 h by infection with the NB tropic FB strain of FLV, as described by Dexter *et al.* (1977).

*Isolation and characterisation of a transformed cell line.*—Cells collected 14 days after the infection with FLV were plated in alpha medium plus 30% foetal calf serum, $10^{-7}$M transferrin and $10^{-7}$M sodium selenite (Guilbert & Iscove, 1976) in 0·8% methyl cellulose as described previously (Testa & Dexter, 1977). After 10 days of incubation at 37°C in an atmosphere of 5% $CO_2$ in air, large compact colonies were observed (Testa *et al.*, 1979). They were pooled and subcultured in the same growth medium, but without methylcellulose in culture flasks. These cells, designated 427E, grew in suspension and were subcultured weekly after saturation growth was reached (*i.e.* every 6–7 days). The growth medium harvested before subculturing (CM = conditioned medium) was stored at −20°C until assayed for its activity (colony stimulating activity = CSA) in inducing the formation of granulocyte-macrophage colonies by normal marrow cells.

For subsequent *in vitro* colony assay of 427E cells, these were plated in Petri dishes in Fischer's medium plus 25% horse serum in 0·3% agar, as described previously (Dexter & Testa, 1976). In some experiments, medium conditioned by mouse heart was used as the source of CSA, which is essential for the growth of colonies of granulocytes and macrophages from normal haemopoietic cells. These cultures were incubated at 37°C in a humidified atmosphere of 5% $CO_2$ in air, and colonies of more than 50 cells were scored after 7 days.

For possible induction of leukaemia, 427E cells harvested from suspension cultures, or (in one experiment) from colonies grown in agar, were injected into syngeneic neonatal or adult mice in doses of $10^5 - 5 \times 10^6$ cells per mouse.

Cytogenetic studies were performed 5 weeks after the isolation of the cell line, using standard techniques. In two cases, tissues from leukaemic mice were investigated; marrow and tumour tissue preparations were made according to the methods of Ford (1966) and Evans *et al.* (1972) respectively.

The infectivity of the lymphatic leukaemia virus (LLV) complex was measured using the XC plaque-forming assay of Rowe *et al.* (1970) on NIH/3T3 cells. The spleen focus-forming virus (SFFV) was tested by injecting 0·2 ml of cell-free supernatants into normal mice. The mice were killed 7 days later and their spleens were examined for the presence of foci.

### RESULTS

*Growth characteristics of 427E cells.*—The cells isolated as described above from colonies derived from FLV-infected long-term marrow cultures grow in suspension with a doubling time of 12–16 h in the absence of the adherent cell layer which is necessary for the maintenance of prolonged haemopoiesis *in vitro* (Dexter & Lajtha, 1974). The cultures reach plateau phase at concentrations of the order of $1\text{–}2 \times 10^6$ cells per ml. Morphologically 20–40% of the cells are classified as blasts, and a similar proportion as promyelocytes. Few late granulocytes or mononuclear cells are observed. Table I shows the distribution of cells harvested at different times after the isolation of the 427E cells. Myeloperoxidase-positive cells constituted 3·5 and 8% of the population when investigated at Weeks 5 and 7.

The cells from the suspension cultures produce CSA which stimulates the formation of granulocyte-macrophage colonies by normal marrow cells in a dose-dependent fashion giving a sigmoid dose–response curve. Colony formation starts at 1% concentration and reaches plateau levels of the order of 100 colonies per $10^5$ normal cells when the CM is added at 30% concentration. Both the number of colonies and the morphology of the colony cells were comparable to those obtained using mouse heart CM as the source of CSA.

When plated in soft agar, the 427E cells form colonies with a plating efficiency of 10–15%, and occasionally as high as 30%. There is a linear relationship between number of colonies and number of cells plated. The numbers of colonies are not influenced by the addition of exogenous CSA, even at very low cell inocula (Fig. 1). The colonies are spherical and compact, and contain up to $5 \times 10^3$ cells which exhibit little sign of differentiation after a week of culture in soft agar. Blast cells and early granulocytes represent 90% of the total colony cells, with the rest composed of late granulocytes and mononuclear cells. The shape and composition of the colonies

TABLE I.—*Morphological identification of 427E cells in liquid culture*

| Weeks in culture | Cells/ml × $10^{-5}$ | Morphology (%) Blasts | Early granulocytes | Late granulocytes | Mononuclears |
|---|---|---|---|---|---|
| 4 | ND | 36 | 42 | 14 | 6 |
| 5 | 4·8 | 22 | 71 | 6 | 0 |
| 6 | 11·0 | 42 | 56 | 1 | 0 |
| 12 | 7·4 | 38 | 60 | 1 | 0 |
| 40 | 7·6 | 59 | 39 | 0 | 2 |

ND = Not done.
Early granulocytes = Promyelocytes, myelocytes.
Late granulocytes = Metamyelocytes, segmented granulocytes.

are similar when the 427E cells are grown for 7 days in the presence of 15% mouse heart CM, a dose which is in the plateau zone for the induction of colony formation by normal marrow cells. However, in spite of this apparent lack of effect, the CM decreased the plating efficiency of 427E cells from individual colonies from 451 ± 69 colonies per replated colony (when the original colonies were grown in the absence of CM) to 96 ± 24 when cells from colonies grown in the presence of CM were replated.

Most of the cultured cells examined have 78 chromosomes, including 2 large metacentrics which may have arisen as a result of a Robertsonian translocation between two acrocentrics (Robertson, 1916) in which case these cells can be regarded as being tetraploid (Fig. 2). Cells with 72–78 chromosomes were also found, as well as some cells (< 10% of the total examined) with very high chromosome numbers (~ 250). No cells had a diploid or near diploid constitution.

FIG. 2.—Metaphase preparation of 427E cell in culture, with 2 metacentric chromosomes (arrowed).

FIG. 1.—Formation of colonies in agar by 427E cells in the presence (●) or absence (×) of exogenous CSA.

TABLE II.—*Friend leukaemia virus replication in cultures of 427E cells**

| Weeks in culture | LLV component (PFU/ml) Original FLV infected marrow culture | 427E cells |
|---|---|---|
| 1 | 2·2 × $10^3$ | ND |
| 3 | 2·2 × $10^3$ | 3·4 × $10^4$ |
| 5 | ND | 1·4 × $10^4$ |
| 15 | 3·0 × $10^5$ | 4·2 × $10^5$ |

* The SFFV component was not detected in either the original marrow culture or the 427E cells when investigated at 1, 3 or 5 weeks of culture.
ND = Not done.

TABLE III.—*Leukaemia-inducing ability of* 427*E cells*

| Mice | No. of leukaemic mice/No. inoculated | No. of cells injected (route of inoculation) | Day of killing or death | Tumours at the site of injection | Infiltration of haemopoietic tissue |
|---|---|---|---|---|---|
| Neonatal | 2/2 | $2 \times 10^6$ (s.c.) | 22–34 | Yes | Yes |
| | 4/4 | $10^6$ (s.c.) | 19–34 | Yes | Yes |
| | 2/2 | $10^6$ (i.p.) | 19–41 | Yes | Yes |
| Adult | 3/4 | $5 \times 10^6$ (i.v.) | 35–96 | NA | Yes |
| | 2/2 | $\sim 10^5$ (s.c.)* | 37–45 | Yes | Yes |

* Cells from 25 colonies grown in soft agar (see text).
NA = not applicable.

Replication of LLV occurs in the 427E cells at comparable levels to those observed in the original marrow culture (Table II). SFFV, however, has not been detected in either culture, by titrating spleen foci or by measurement of erythroleukaemia development. Consequently it is not surprising that neither the cells nor the cell-free supernatant had the ability to produce erythroleukaemia when injected into mice.

*Induction of leukaemia.*—The results in Table III indicate that the injection of 427E cells into syngeneic neonatal or adult mice induces an acute leukaemia which reaches an advanced state at the times indicated. Infiltration of haemopoietic tissue and liver by malignant cells was always found. In addition, where the cells were administered either s.c. or i.p., the tumour mass at the site of injection often reached 7–12 mm in diameter in neonatal mice, or up to 32 mm in adults. Chromosome analysis was performed in cells from one tumour (from mouse no. 4 in Table IV) which showed that 90% of the cells were tetraploid or hypotetraploid with the same 2 metacentric marker chromosomes as in the cultured cells (Fig. 2). Similar analysis of one marrow sample (mouse no. 1 in Table IV) showed that 25% of the cells had a similar chromosome constitution, whereas the rest had a normal karyotype.

Histological examinations of sections of tumour tissue showed that, in contrast to

TABLE IV.—In vitro *colony formation by cells from leukaemic mice*

| Recipients | Mouse No. | No. of 427E cells administered (route of inoculation) | Day of sampling | Exogenous CSA | Colonies per $10^5$ cells: Marrow | Spleen | Tumour |
|---|---|---|---|---|---|---|---|
| Neonatal | 1 | $2 \times 10^6$ (s.c.) | 22 | + | 4200 | 126 | 380 |
| | | | | – | 3962 | 123 | 377 |
| | 2 | $10^6$ (s.c.) | 34 | + | 1120 | 205 | 242 |
| | | | | – | 1368 | 158 | 168 |
| | 3 | $10^6$ (i.p.) | 37 | + | 66 | 12 | ND |
| | | | | – | 15 | 1 | ND |
| | 4 | $10^6$ (i.p.) | 41 | + | 225 | ND | > 1000 |
| | | | | – | 455 | ND | > 1000 |
| Adult | 5 | $\sim 10^5$ (s.c.)* | 38 | + | 288 | 36 | 1562 |
| | | | | – | 12 | 0 | 861 |
| | 6 | $5 \times 10^6$ (i.v.) | 31 | + | > 5000 | ~ 1600 | NA |
| | | | | – | > 5000 | ~ 1600 | NA |
| | 7 | $5 \times 10^6$ (i.v.) | 31 | + | > 5000 | ND | NA |
| | | | | – | > 5000 | ND | NA |

* Cells from colonies grown in soft agar (see Table III).
ND = not done.
NA = not applicable.

the little degree of differentiation shown in suspension cultures or in agar colonies, a large proportion of the cells were metamyelocytes and segmented granulocytes. However, undifferentiated cells were seen in marrow, spleen and liver (data not shown).

Cells from the marrow, spleen or tumour from some leukaemic mice were assayed for their capacity to form colonies in soft agar. All samples assayed showed colony formation in the absence of exogenous CSA, in some cases with a plating efficiency as high as 5% (Table IV). Tissues from 2 mice showed low numbers of colonies which could be increased by the addition of CSA. However, some of the colonies in cultures with exogenous CSA had a normal appearance. These were probably derived from normal granulocyte-macrophage progenitor cells still in the haemopoietic organs.

### DISCUSSION

It is known that normal granulopoiesis can be stimulated by *in vivo* or *in vitro* infection with FLV (Golde *et al.*, 1976; Dexter *et al.*, 1977). *In vitro* infection of freshly isolated foetal liver or adult marrow with FLV may produce cell lines which show erythroid characteristics after treatment with dimethyl sulphoxide (Golde *et al.*, 1979; Revoltella *et al.*, 1979) and which may induce local tumours resembling reticulum-cell sarcomas upon inoculation into mice. We have now shown that FLV may also induce leukaemic granulopoiesis.

The early loss of the SFFV component may be critical in determining the pattern of differentiation after FLV treatment. When both components replicate *in vitro*, injection of cells or cell-free supernatant from infected marrow cultures causes erythroleukaemia of host origin (Dexter *et al.*, 1977). Replication of only the LLV component may lead to the production of multipotential cell lines that differentiate normally into granulocytes and megakaryocytes, and which are not leukaemogenic (Dexter *et al.*, 1979) or, as in the present studies, to the production of cells with the potential of producing myelomonocytic leukaemia.

In contrast to the usual latent period of several weeks before changes in the differentiation or proliferation capacity of cells in infected cultures can be detected (Dexter *et al.*, 1977) the 427E cells were isolated 14 days after FLV treatment. Whether the early detection of leukaemic transformation was because the cells were allowed to proliferate in the absence of the adherent cell layer which is necessary for the maintenance of normal haemopoiesis *in vitro*, is not known at present.

The predominantly near-tetraploid constitution of the 427E cell line is atypical. Previous studies on FLV-transformed haemopoietic cells have demonstrated a diploid or near-diploid constitution (Ostertag *et al.*, 1972; Elliot *et al.*, 1972; Dexter *et al.*, 1977; Revoltella *et al.*, 1979); therefore some of the unusual properties of this cell line may be attributed to its increased ploidy. It is unlikely that the presence of the metacentric chromosomes is related to the malignancy of this cell line, since, although there are several reports of FLV-transformed cells carrying metacentric chromosomes (Ostertag *et al.*, 1972; Golde *et al.*, 1979; Revoltella *et al.*, 1979) such chromosomes were not seen by Elliot *et al.* (1972) or by Dexter *et al.* (1977) in FLV-induced leukaemias. However, the presence of the 2 metacentric markers in tetraploid and hypertetraploid cells of tumour tissue and marrow of mice inoculated with 427E cells provides good evidence of the donor origin of the leukaemias.

Only a small number of myeloid leukaemias have been described in mice. The available information has recently been reviewed by Metcalf (1977). Most of these leukaemias have been unable to proliferate in agar when first isolated. When able to do so, the addition of CSA increased the plating efficiency and induced morphological differentiation (Ichikawa, 1969; Metcalf *et al.*, 1969). The behaviour of the 427E cells differs from that described: they show high plating efficiency, independence of exogenous CSA for colony formation, even at very low cell inocula, and the cells do not appear to differentiate further after its addition. However, the decrease in plating efficiency of cells from colonies grown with exogenous CSA suggest that they may conserve some response to it. These cells may be able to mature further when inoculated into mice. The high proportion of cells with the chromosome markers in the tumour tissue examined, together with the low proportion of differentiated granulocytes in the infiltrated haemopoietic tissue, and the observation that the frequency of colony-forming cells may be higher in the marrow than in localized tumours, support the concept that the tumour site may provide an environment more favourable to differentiation than the marrow. However, the possibility that the granulocytes found in the tumour tissue were reactive host cells can not be definitely ruled out at present. The influence of microenvironmental factors in modulating cell maturation in leukaemic cells (Metcalf & Moore, 1970) is shown by the different proportions of relatively mature granulocytes in various tissues of these leukaemic mice. Unlike WEH1-3 cells grown in subcutaneous or intraperitoneal tumours (Metcalf & Moore, 1970) 427E cells conserve high clonogenic capacity in those circumstances. It is interesting to note that, as is the case in chronic myeloid leukaemias in humans, the high clonogenic capacity is observed in spite of the production of large numbers of more differentiated cells.

Further study of the conditions which influence changes in clonogenic and leukaemogenic capcity, and investigation of the role of CSA and host factors in the modulation of cell maturation, should increase our understanding of the behaviour of leukaemic cells.

The technical assistance of Ms M. Booth and G. Johnson is gratefully acknowledged. This work was supported by grants from the Medical Research Council and the Cancer Research Campaign.

### REFERENCES

DEXTER, T. M. & LAJTHA, L. G. (1974) Proliferation of haemopoietic stem cells *in vitro*. *Br. J. Haematol.*, **28,** 525.

DEXTER, T. M. & TESTA, N. G. (1976) Differentiation and proliferation of haemopoietic cells in culture. In *Methods in Cell Biology*. Vol. 14, Ed. D. M. Prescott. New York: Academic Press. p. 387.

DEXTER, T. M., SCOTT, D. & TEICH, N. M. (1977) Infection of bone marrow cells *in vitro* with FLV: Effects on stem cell proliferation, differentiation and leukaemogenic capacity. *Cell*, **12,** 355.

DEXTER, T. M., ALLEN, T. D., SCOTT, D. & TEICH, N. M. (1979) Isolation and characterisation of a bipotential haemopoietic cell line. *Nature*, **277,** 471.

ELLIOT, S. C., HELM, R. M. & MYSZEWSKI, M. E. (1972) Sequential changes in spleen cell chromosomes during Friend virus leukaemia. *Cancer Res.*, **32,** 776.

EVANS, E. P., BURTENSHAW, M. D. & FORD, C. E. (1972) Chromosomes of mouse embryos and newborn young: Preparations from membranes and tail tips. *Stain Technol.*, **47,** 229.

FRIEND, C. (1957) Cell-free transmission in adult Swiss mice of a disease having the character of leukaemia. *J. Exp. Med.*, **105,** 307.

FORD, C. E. (1966) The use of chromosome markers. In *Tissue Grafting and Radiation*. Eds H. S. Micklem & J. F. Loutit. New York: Academic Press. p. 197.

GOLDE, D. W., FAILLE, A., SULLIVAN, A. & FRIEND, C. (1976) Granulocytic stem cells in Friend leukaemia. *Cancer Res.*, **36,** 115.

GOLDE, D. W., BERSCH, N., FRIEND, C., TSUEI, D. & MAROWITZ, W. (1979) Transformation of DBA/2 mouse fetal liver cells infected *in vitro* by the anemic strain of Friend leukaemia virus. *Proc. Natl Acad. Sci. USA*, **76,** 962.

GUILBERT, L. J. & ISCOVE, N. N. (1976) Partial replacement of serum by selenite, transferrin, albumin and lecithin in haemopoietic cell cultures. *Nature*, **263,** 594.

ICHIKAWA, Y. (1969) Differentiation of a cell line of myeloid leukemia. *J. Cell Physiol.*, **74,** 223.

METCALF, D. (1977) *Hemopoietic colonies*. Berlin: Springer-Verlag.

METCALF, D. & MOORE, M. A. S. (1970) Factors modifying stem cell proliferation of myelomonocytic leukaemic cells *in vitro* and *in vivo*. *J. Natl Cancer Inst.*, **44,** 801.

METCALF, D., MOORE, M. A. S. & WARNER, N. L. (1969) Colony formation *in vitro* by myelomonocytic leukemic cells. *J. Natl Cancer Inst.*, **43,** 983.

OSTERTAG, W., MELDERIS, H., STEINHEIDER, G., KLUGE, N. & DUBE, E. (1972) Synthesis of mouse haemoglobin and globin m-RNA in leukaemic cell cultures. *Nature* (*New Biol.*), **239,** 231.

REVOLTELLA, R., BERTOLINI, L. & FRIEND, C. (1979) *In vitro* transformation of mouse bone marrow cells by the polycythemic strain of Friend leukaemia virus. *Proc. Natl Acad. Sci USA*, **76,** 1464.

ROBERTSON, W. R. B. (1916) Chromosome studies: Taxonomic relationships shown in the chromosomes of Tettigidae and Acrididae: V shaped chromosomes and their significance in Acrididae, Locustidae, Gyrillidae: Chromosomes and variations. *J. Morphol.*, **27,** 179.

ROWE, W. P., PUGH, W. E. & HARTLEY, J. W. (1970) Plaque assay techniques for murine leukemia viruses. *Virology*, **42**, 1136.

TAMBOURIN, P. E. & WENDLING, F. (1975) Target cell for oncogenic action of polycythaemia-inducing Friend virus. *Nature*, **256**, 320.

TESTA, N. G. & DEXTER, T. M. (1977) Long-term production of erythroid precursor cells (BFU) in bone marrow culture. *Differentiation*, **9**, 193.

TESTA, N. G., DEXTER, T. M. & TEICH, N. M. (1979) Characterisation of myelomonocytic leukaemia cells indiced by *in vitro* infection of bone marrow with FLV. In *Modern Trends in Human Leukaemia III* (Eds Neth *et al.*). Verlag. p. 231.

### ADDENDUM

Since the completion of this work Greenberger *et al.* (1979: *Blood*, **53**, 987) reported the induction of promyelocytic leukaemic cells in marrow cultures infected with FLV-A.